# What is healthcare for?

**DOI:** 10.1080/17571472.2015.1113715

**Published:** 2015-12-24

**Authors:** Peter Toon

## Why this matters to me

*I find the lack of clarity about the fundamental purpose of health care which causes moral conflict and inappropriate clinical activity, damaging clinicians and patients alike, very disturbing.*

## What is medical ethics?

If you read the newspapers or listen to Radio 4 you would get the impression that medical ethics is the discussion of moral problems posed by modern medical technology or bizarre situations – whether and when it is permissible to turn off life support machines [1], the implications of ever more sophisticated techniques in assisted conception [2] or whether to amputate a normal leg because the patients asks for it [3]. This I call ‘tabloid ethics’.

If you work in primary health care however you will know that most ethical problem which practitioners face are not caused by cutting edge technology or extraordinary situations, but by the normal messy complexities of human life and relationships. Exactly what do you write and how do you phrase it when asked about a health problem in a medical report which may affect someone’s insurance, mortgage or job?[4] How hard should you try (if at all) to persuade someone with a high cholesterol of the benefits of statins? How do you handle information which you obtain from one person in confidence which has implications for the health or well-being of someone else?

Although they still do not get the attention they deserve, there is increasing recognition within the health professions, if not in the media, of the importance of these and the many other questions which make up ‘everyday ethics’ [5] or ‘the ethics of the ordinary’ [6], and there are conferences, workshops, books, journal articles and websites [7] which try to help practitioners deal with them.

However, a more fundamental question even more rarely discussed underlies both tabloid and everyday ethics. What is health care for? We spend lots of time and money making sure our practice is as efficient and evidence based as possible, but rarely stop to consider why we do what we do.

Many people would say the answer was obvious: saving lives and preventing or relieving pain and suffering. But lives are never saved. Mortality remains 100%, and death is merely postponed. An expression originally used to refer to saving someone from drowning or other sudden traumatic death [8] which can allow a life to be lived into old age which otherwise might end in childhood or youth has come to be used of any intervention which extends life, even if only by a few years or even months.

The view that the purpose of health care is avoiding pain and extending life assumes an implicit consequentialist ethic. What is right is the action which promotes the greatest good of the greatest number, good being seen as the longest possible life with the minimum amount of suffering. This value is operationalised in the ‘Quality-adjusted life year’ (QALY) [9] used by NICE and other bodies to decide which treatments should and should not be funded by the NHS.

But we do not follow this principle through logically. We do not, for example, insist that those with extensive, severe injuries or brain death with little hope of a life with many QALYs should be used as organ banks for those on the transplant list, even though their QALY prospects are much better. The NHS Constitution [10] lists numerous rights of patients on matters such as choice, access to services and confidentiality even though the cost of offering some of these may reduce the amount of good for the greatest number which can be done with the money available to the NHS. We recognise that human dignity limits what we can do to people even if it might promote an overall greater good. We acknowledge human rights both claim rights [11] – things we are entitled to whether the benefits are large or small – and liberty rights – freedoms that must not be curtailed even in a good cause. Our moral framework is further complicated by other perspectives; consumerism, legalism, managerialism, the view of health care as a business. Alisdair MacIntyre argues that we live in a society suffering from moral fragmentation [12] and I have explored how this affects healthcare elsewhere [13].

In ‘*What is Good General Practice*?’[14] I argued that primary healthcare has three elements:

(1) Curing disease or alleviating suffering by biomedical intervention.
(2) Preventing disease by biomedical intervention.
(3) Helping people understand and make sense of their illnesses – the interpretative function.

The first two of these functions fits well with the view that the purpose of health care is maximising life and minimising suffering, albeit with the restrictions imposed by human rights and the other factors discussed above. The third however implies a very different view of the purpose of health care and indeed of life itself. People come to doctors and nurses not solely or even mainly in order have their pain alleviated and their lives lengthened, but to understand what is happening to them and to make sense of it within their overall understanding of the nature and purpose of their lives; their ‘narrative identity’ or ‘personal life narrative’ [15]. This implies that we are not merely looking for a life which is the longest string of pleasurable experiences possible with the minimum number of unpleasant ones, but for a life which makes sense and has a purpose and a shape. We are seeking a narrative of flourishing.

The personal view from a medical student in this edition describes a very clear example of this type of work, when a GP sees a patient facing a difficult decision about cancer treatment to help her move towards a narrative which will help her to flourish. The surprise the author reports on seeing this work in action illustrates how poor we are at communicating that this is a vital element in our work.

Flourishing (in Greek *eudaemonia* [16], literally having a good soul or spirit) is the central concern of ancient and medieval ethics but rather disappeared from philosophy after the Enlightenment. There has however been a renaissance of interest in what it means and how to achieve it (an approach usually known as ‘virtue ethics’) in recent years. This approach to ethics focuses not on what rules we should follow or how to maximise pleasure but how to live so as to have full, meaningful lives. This change of emphasis in philosophy has been accompanied by a parallel shift in psychology, towards the study of happiness rather than psychopathology, sometimes called ‘positive psychology’ [17].

Philosophers from Aristotle [18] onwards have argued that the route to flourishing is to cultivate the virtues. This does not mean seeking saintly perfection (though a few may see that as their goal in life), but rather acquiring those personal qualities which help us to overcome the challenges life throw as at us to a sufficient degree to enable us to survive them and to grow through them.

For health care professionals many of those challenges happen at work, and virtue ethics has much to offer health care. Unlike other approaches to ethics, it is holistic, addressing not only how we think rationally in tricky situations but also what emotions and motivations help us to do the right thing, and how this is affected by our bodily well-being as well as our minds. It provides us with a rationale for what we do; through helping our patients to flourish we also lead more flourishing lives. In a world where professional morale is low and burnout common [19] this is a major benefit.

It provides a clear rationale for professionalism and helps us define it; health care professionalism is the sum of the qualities which one needs to flourish as a health professional. It has implications for how health care is organised and for the institutions which support and govern it – structures which often seem confused and under strain at present.

Perhaps most importantly it helps solve the central paradox of health care. If we see our purpose solely as postponing death and alleviating pain then ultimately we are doomed to failure, since death (like taxes) is ultimately inevitable. Our attempts to ‘save lives’ often succeed merely in replacing death from one cause with death a little later from another, and the later death may involve a long slow decline in which both life and death cease to have any meaning.

As I have argued at much greater length elsewhere,[20, 21] I think that a virtue ethic offers the most satisfactory answer to the question of what healthcare is for. But whether or not I am right, this is a issue which deserves far more attention than it currently receives. We need a public discussion on the fundamental purpose of health care; a discussion which acknowledges not only that we cannot afford to do all the things we might do to prolong life, but also that even could we do so in the end we will die. And this discussion needs to take a global view of health care, rather than focus on specific illnesses or problems such as cancer or dementia. Patients, clinicians, managers and health care students all need to think about this question if we are to have a health service which is both rational and evidence based.

If we just spend time thinking about systems of care or isolated moral problems without considering this central question of medical ethics, we will be like travellers deciding which is the best maintained road and which choice we should make at a cross roads without knowing where we are trying to go to.

Peter D. Toon July 2015
